# Kaposiform lymphangiomatosis and kaposiform hemangioendothelioma: similarities and differences

**DOI:** 10.1186/s13023-019-1147-9

**Published:** 2019-07-05

**Authors:** Yi Ji, Siyuan Chen, Suhua Peng, Chunchao Xia, Li Li

**Affiliations:** 1 Division of Oncology, Department of Pediatric Surgery, West China Hospital of Sichuan University, Chengdu, 610041 China; 2 Pediatric Intensive Care Unit, Department of Critical Care Medicine, West China Hospital of Sichuan University, #37# Guo-Xue-Xiang, Chengdu, 610041 China; 3 Department of Radiology, West China Hospital of Sichuan University, Chengdu, 610041 China; 4 Laboratory of Pathology, West China Hospital of Sichuan University, Chengdu, 610041 China

**Keywords:** Kaposiform lymphangiomatosis, Kaposiform hemangioendothelioma, Thrombocytopenia, Coagulopathy, Thorax

## Abstract

**Background:**

Kaposiform lymphangiomatosis (KLA) and kaposiform hemangioendothelioma (KHE) are rare and aggressive vascular disorders. The aim of this study was to examine the clinical features and prognosis of KLA and KHE involving the thorax.

**Methods:**

The clinical features, imaging and pathological findings, treatments and outcome were retrospectively reviewed for 6 patients with KLA and 7 patients with KHE involving the thorax.

**Results:**

The mean ages at the time of the presentation of signs/symptoms were 26.7 months and 4.1 months for KLA and KHE, respectively. Respiratory symptoms, pericardial and pleural effusion, thrombocytopenia and coagulopathy were common in both KLA and KHE. Diffuse lesions involving the lung and extrathoracic sites were observed in KLA but not in KHE. Histopathologically, all lesions had spindled tumor cells, which were immunoreactive for CD31 and D2–40. In KLA, the spindle cells were distributed in sparse and poorly marginated clusters, whereas the spindle cells formed more defined and confluent vascularized nodules in KHE. Unlike the refractory behavior of KLA, the majority of patients with KHE responded to medical treatments with regression of the lesion and normalization of the hematologic parameters.

**Conclusions:**

The presenting and histological characteristics of KLA can overlap with those of KHE. The presence of diffuse vascular lesions in the mediastinum and lung with refractory thrombocytopenia and coagulopathy should suggest the diagnosis of KLA. Given the rarity and high morbidity and mortality of these disorders, the diagnostic process and therapeutic approach should include a multidisciplinary team review and consensus.

**Electronic supplementary material:**

The online version of this article (10.1186/s13023-019-1147-9) contains supplementary material, which is available to authorized users.

## Introduction

Kaposiform lymphangiomatosis (KLA) and kaposiform hemangioendothelioma (KHE) are rare vascular anomalies, both of which have locally aggressive characteristics. These two types of vascular anomalies can present at birth or shortly after birth and are associated with high morbidity and mortality [1–3]. KLA can be localized to the mediastinum and lung, or most often, can be widespread with the involvement of multiple extrathoracic organs. KHE is found in varied locations, but there is a predilection for the extremities and trunk. Clinically, KLA and KHE are often associated with life-threatening thrombocytopenia and consumptive coagulopathy. Histopathologically, KLA and KHE are characterized by spindled endothelial cells and malformed lymphatic channels [2, 4]. The natural histories of these disorders and the appropriate treatment regimen remain controversial.

KHE involving the thorax is extremely rare, with few cases reported in the literature [5–7]. However, KHE involving the thorax can share overlapping characteristics in terms of clinical symptoms (e.g., respiratory distress), anatomical location, imaging findings and complications (e.g., pleural and pericardial effusions) with KLA. Given the rarity, clinical similarities and sometimes confusing histopathologic findings between KLA and KHE, these diseases can be misclassified or overlooked. Therefore, the objective of our study was to describe and analyze the characteristics of these vascular disorders. More precise recognition of the distinct features of KLA and KHE will facilitate their diagnosis and the prompt application of pharmacologic interventions.

## Methods

Institutional review board approval for this retrospective study was obtained from the West China Hospital of Sichuan University. All procedures followed the research protocols approved by West China Hospital of Sichuan University and Sichuan University and were conducted according to the Declaration of Helsinki. Written informed consent was obtained from the parents of all patients.

The study included patients with KLA and patients with KHE involving the thorax. All patients were diagnosed and followed at the West China Hospital of Sichuan University over a 15-year period between January 2003 and December 2017. The diagnoses of KLA and KHE were based on clinical presentation/features, imaging studies and/or histological data [2, 8, 9]. Based on the findings of computed tomography (CT) scans and/or magnetic resonance imaging (MRI), only KHEs with thoracic involvement were included. Part of the KHE cohort was included in our previous study [4]. Patients were excluded if they had insufficient data.

Data obtained from the medical records included demographics, clinical presentation, associated complications, laboratory results, imaging results, treatment, follow-up examinations and outcome. According to previous studies, the Kasabach-Merritt phenomenon (KMP) in KHE was defined as a platelet count less than 100 ×  10^9^/L with consumptive coagulopathy and hypofibrinogenemia. Severe acquired hypofibrinogenemia was defined as a fibrinogen (FIB) level less than 1.0 g/L. Severe anemia was defined as a hemoglobin concentration less than 80 g/L [10]. Elevated D-Dimer was defined as a test result more than 500 μg/L. Prolonged prothrombin time (PT) and active partial thromboplastin time (APTT) were defined as values above 15 and 40 s, respectively. Associated chronic pain was defined as daily pain for at least 3 months. Response to therapy was classified as successful, stabile and worse. Based on previous studies, a successful treatment response was defined as an improvement in the patient’s symptoms and/or complications, the restoration of hematologic parameters, and a reduction in the lesion volume [11].

## Results

### Baseline characteristics

Thirteen patients were included in the study. A summary of the patients is presented in Table 1. For additional details regarding the clinical characteristics of the patients see the Additional file 1: Table S1. Of these 13 patients, six were diagnosed with KLA. The mean age at diagnosis of KLA was 52.8 months (range 13.0 to 108.0 months). There were four males and two females. Two patients initially presented with non-productive cough and fatigue. These patients had diffuse pulmonary opacities on chest radiography and were suspected to have a pulmonary infection. Multiple courses of antibiotic therapy were administered, but their condition did not improve after treatment. Another two patients were first admitted to local hospitals for increasing signs of respiratory distress. The remaining two patients did not have respiratory signs or symptoms; one presented with abdominal distention and vomiting, and the other presented with progressive cutaneous bruising and a subcutaneous mass.

**Table 1:** Demographic and Clinical Characteristics of patients with KLA and KHE ^a^

Variables	KLA	KHE
	*n* = 6	*n* = 7
Patients		
Gender^b^		
Male	2 (33.3)	2 (28.6)
Female	4 (66.7)	5 (71.4)
Age at the time of the presentation of the signs and/or symptoms (m)		
Mean (range)	26.7 (8.0–72.0)	4.1 (0.0–10.0)
Age at diagnosis (m)		
Mean (range)	52.8 (13.0–108.0)	6.5 (0.3–14.0)
Vascular lesions		
Involvement^b^		
Skin	1 (16.7)	4 (57.1)
Lung	5 (83.3)	0 (0.0)
Bones	4 (66.7)	4 (57.1)
Peritoneal cavity	3 (6.3)	0 (0.0)
Manifestation^b^		
Focal	0 (0.0)	6 (85.7)
Multifocal	0 (0.0)	1 (14.3)
Diffuse	6 (100.0)	0 (0.0)
Follow-up (y)		
Mean (range)	4.8 (3.2–6.0)	4.3 (1.2–9.0)
Outcome^b^		
Improved	1 (16.7)	6 (85.7)
Stable	1 (16.7)	0 (0.0)
Worse	2 (33.3)	0 (0.0)
Death	2 (33.3)	1 (14.3)

^a^*KLA* kaposiform lymphangiomatosis, *KHE* kaposiform hemangioendothelioma;

^b^Values are presented as the number of cases (percentage)

KHE was reported in 7 patients, including 5 males and 2 females. The median age at the time of the presentation of signs and/or symptoms was 4.1 months (range 0.0 to 10.0 months). The median age at diagnosis of KHE was 6.5 months (range 0.3 to 14.0 months). Four patients initially presented with cutaneous abnormalities that ranged from an erythematous papule, plaque, or nodule to an indurated firm purple mass. In these four patients, cutaneous signs proceeded tumor recognition in 2 patients. Another three patients did not have cutaneous involvement. The initial presenting features in these non-cutaneous patients included respiratory symptoms (2/3) and increased listlessness with decreased appetite (1/3).

### Hematological parameters

Routine blood parameters and coagulation function were monitored regularly in all patients. Five patients with KLA had confirmed thrombocytopenia, with a mean lowest platelet level of 41 × 10^9^/L (range 3 to 75 × 10^9^/L) during the diagnostic period; three patients had no evidence of thrombocytopenia at the initial assessment, but thrombocytopenia developed later (Table 2); two patients presented with severe thrombocytopenia at the initial assessment. All patients with KHE sustained KMP. The mean lowest platelet count was 13 × 10^9^/L (range 4 to 26 × 10^9^/L).

**Table 2:** Comparison of hematological parameters at the time of lowest recorded platelet count^a^

Cases	PLT (× 10^9^/L)	HGB (g/L)	WBC (× 10^9^/L)	FIB (g/L)	PT (sec)	APTT (sec)	D-Dimer (μg/L)
*KLA*							
1	30	77	11.2	0.49	10.5	34.3	674
2	3	92	5.9	0.42	16.8	50.2	3329
3	75	83	7.9	1.44	12.2	36.8	820
4	41	58	9.4	0.61	12.7	44.5	5066
5	58	92	15.8	0.75	18.6	64.2	1087
6	215	119	18.2	2.78	12.4	36.8	20
*KHE*							
7	9	105	8.9	< 0.10	27.8	68.9	8215
8	21	88	13.3	0.51	11.0	35.3	1996
9	7	69	23.5	0.40	25.3	51.8	3744
10	7	55	7.2	0.22	30.2	70.2	11,805
11	26	91	15.6	1.67	10.7	47.6	718
12	4	68	6.9	0.57	15.6	56.0	2354
13	17	102	9.3	0.98	12.0	38.6	903

^a^*KLA* kaposiform lymphangiomatosis, *KHE* kaposiform hemangioendothelioma, *PLT* platelet, *HGB* hemoglobin, *WBC* white blood cell, *FIB* fibrinogen, *PT* prothrombin time, *APTT* active partial thromboplastin time

Acquired severe hypofibrinogenemia (a FIB level less than 1.0 g/L) was observed in four patients with KLA and six patients with KHE. Two patients with KLA and three patients with KHE had severe anemia (hemoglobin concentration less than 80 g/L). All patients with KHE presented with increased levels of D-dimer. Increased PT and APTT were common in patients with KHE.

### Imaging features

**Fig. 1:**
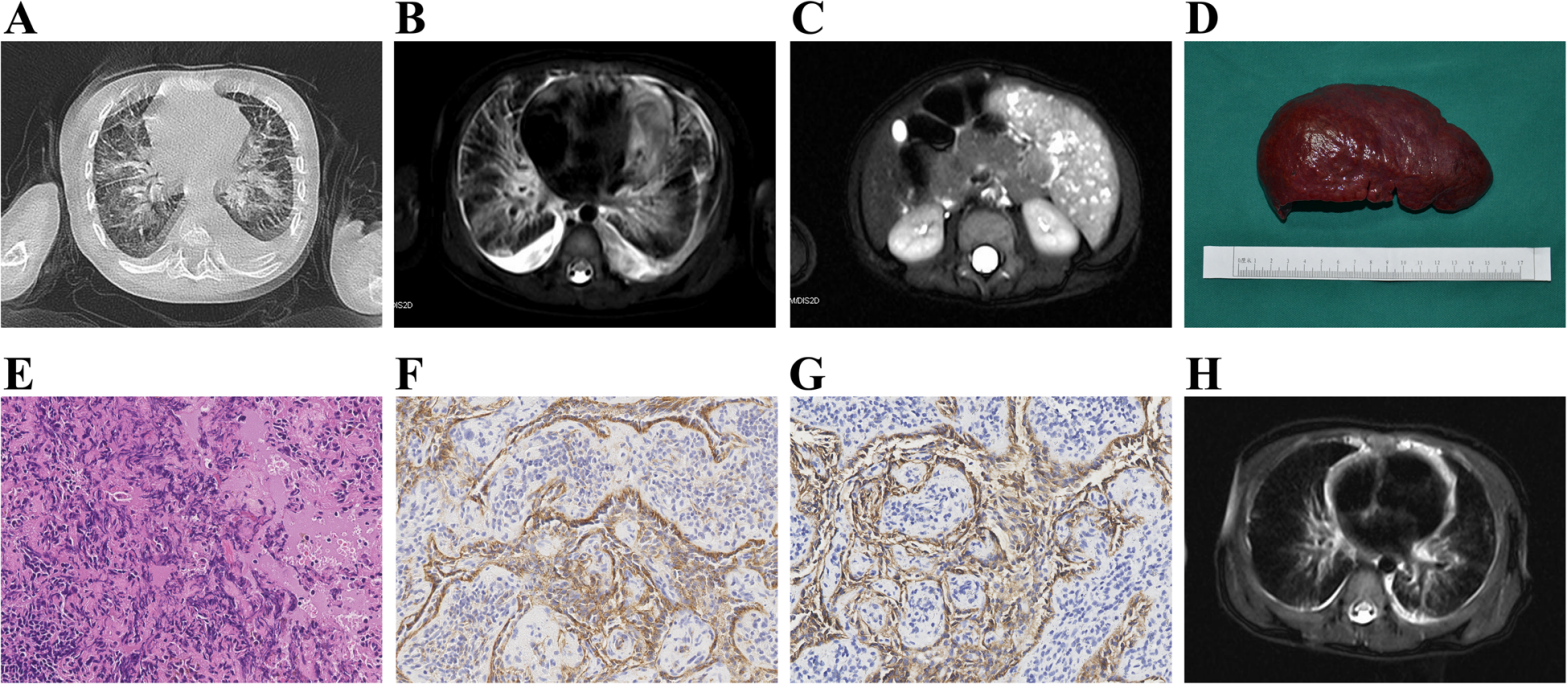
Radiologic findings and pathological features in KLA (Patient #3). **a** Unenhanced CT scan of the chest demonstrates thickening of the interlobular septa. Axial T2-weighted thoracic MRI shows pericardial effusion and high-signal abnormality in the mediastinum that extends along the bronchovascular bundles (**b**). Axial T2-weighted MRI of the abdomen shows multiple high-signal splenic lesions (**c**). Macroscopic view of the spleen excised from the patient (**d**). Multiple, small, reddish blue nodules are noted on the surface of the spleen. Hematoxylin-eosin staining shows dilated lymphatic channels and dispersed small irregular cellular clusters within the splenic parenchyma. The clusters are comprised of spindle-like cells without distinct lumens **e**. The spindle-like cells are immunopositive for CD31 (F) and D2–40 **g**. 6 months after splenectomy, MRI revealed a significant involution of the lesion **h**

**Fig. 2:**
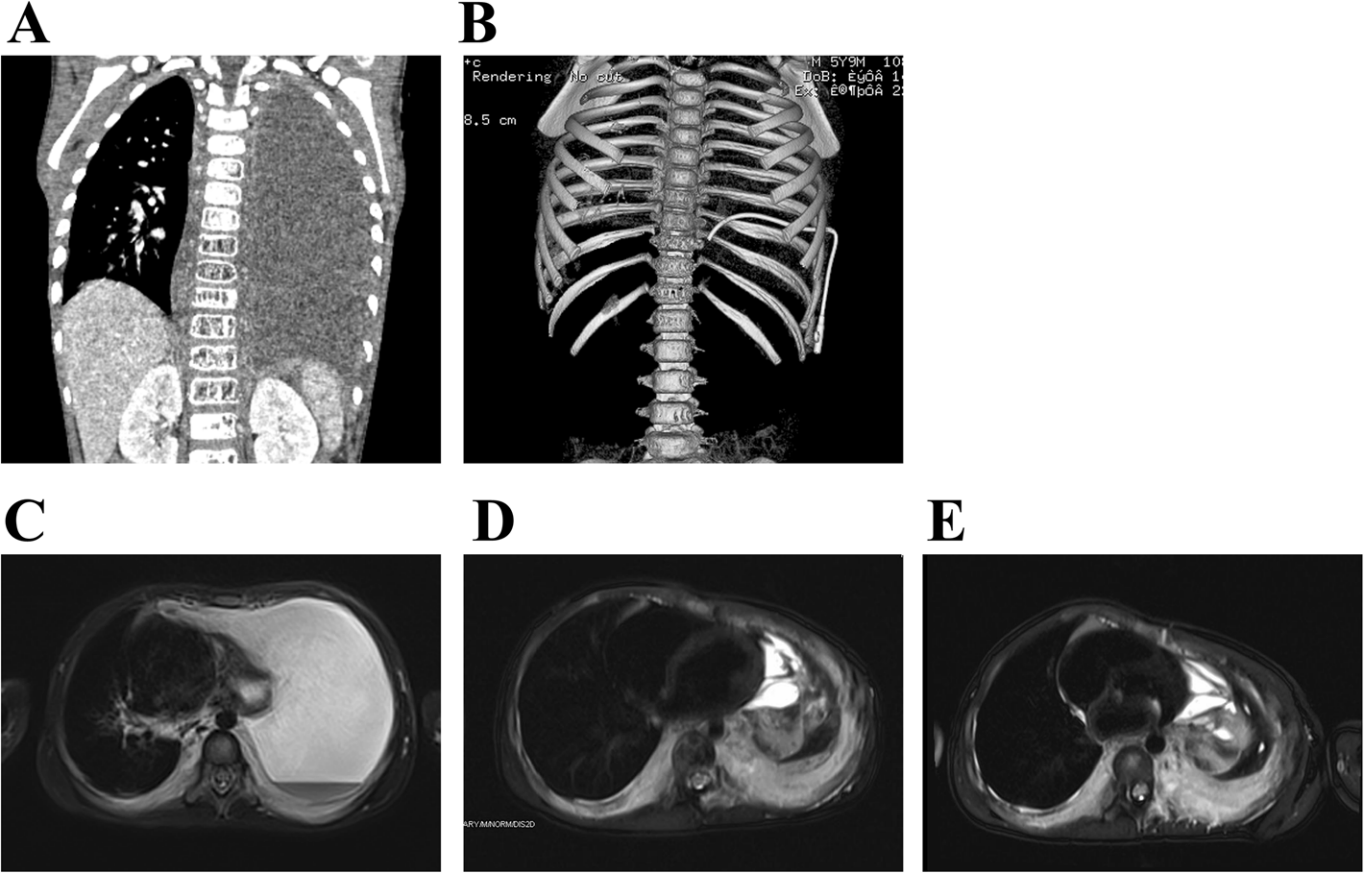
Treatment response in patient with KLA (Patient #1). **a** At the initial presentation of respiratory distress, coronal enhanced CT scan revealed massive pleural effusion almost entirely filling the left hemithorax with complete atelectasis of the left lung and mediastinal shift to the right. The lesions involved the vertebral bodies of T8-T12 and multiple ribs (**b**). Horizontal T2-weighted MRI revealed pleural thickening and effusion, heterogeneously increased T2 signal intensity along the right tracheobronchial tree, and infiltrative, hyperintense, posterior mediastinal soft tissue masses (**c**). The patient was treated with vincristine for 12 months. However, the response was suboptimal. The lesion became progressively static in size (**d**). Treatment with sirolimus and prednisolone was then initiated. After 8 weeks of combination treatment with sirolimus and prednisolone, followed by 10 months of sirolimus monotherapy, the lesions became more prominent (**e**)

CTs and/or MRIs were available for all patients. All patients with KLA had mediastinal involvement. Five patients with KLA had diffuse lesions involving the lung. On thoracic CT scan, the typical imaging finding of KLA was infiltrative soft-tissue thickening (Fig. 1a). MRI showed extensive bilateral ground-glass opacities, thickening of the interlobular septa, and bronchovascular bundles (Fig. 1b). Moderate to severe pericardial effusions (Fig. 1b), pleural effusions (Fig. 2a) and pleural thickening (Fig. 2c) were prevalent. Multiple splenic vascular lesions were identified in two patients (Fig. 1c), including one with lesions simultaneously involving the mesentery, pancreas and liver. Bone invasion or destruction was common (4/6); vertebrae destruction (3/6) and invasion of multiple ribs (2/6) or the pelvis (1/6) was observed (Fig. 2b). One patient (1/6) had lesions involving the ilium, ischium and femur.

**Fig. 3:**
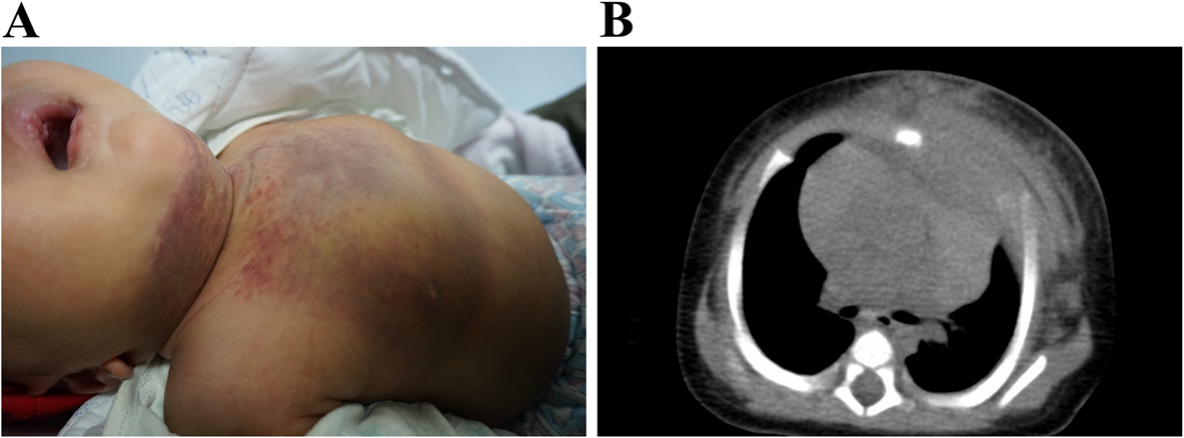
KHE with cutaneous involvement. **a** The photograph shows an indurated and purpuric mass in the anterior neck, chest and abdominal wall (Patient #13). Ecchymosis was evident with telangiectasia. **b** An unenhanced CT scan revealed an extensive soft-tissue mass with ill-defined margins throughout the anterior thoracic musculature

**Fig. 4:**
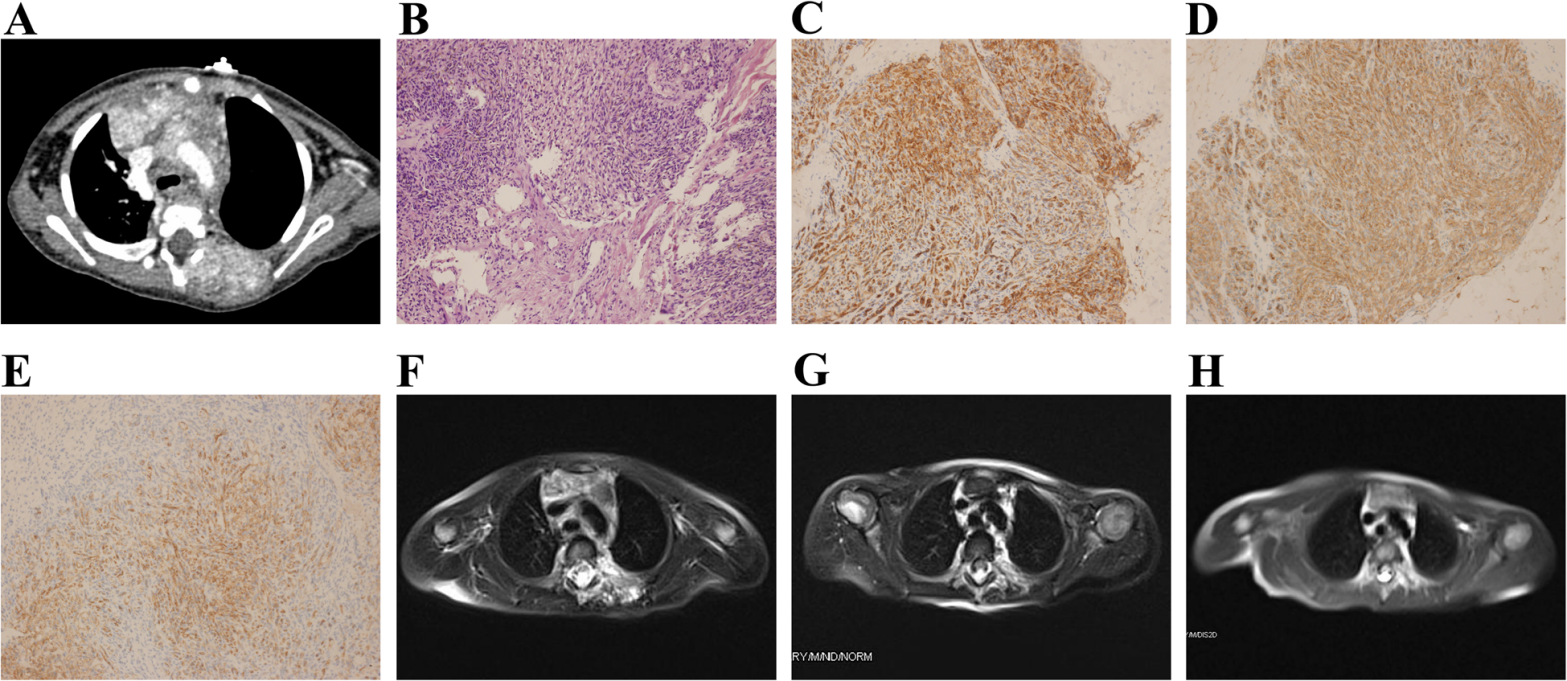
Radiologic findings, pathological features and treatment response in a patient with KHE (Patient #8). **a** An enhanced CT scan shows an enhanced soft-tissue mass extending from the posterior thoracic musculature to the vertebrae. Hematoxylin-eosin staining shows an abnormal proliferation of spindle cells and well-formed capillary-like vessels. Occasional slit-like or round lumens, which were comprised of closely packed spindle cells, could be seen (**b**). The spindle cells were positive for CD31 (**c**), CD34 (**d**), and D2–40 (**e**). The patient received sirolimus in combination with a short-term administration of prednisolone, followed by 22.0 months of sirolimus monotherapy. Prednisolone was successfully tapered and discontinued at 4 and 8 weeks, respectively. MRI showed obvious tumor shrinkage at 6 (**f**), 12 (**g**), and 24 (**h**) months after the start of sirolimus treatment

On unenhanced CT scans, the classic appearance of KHE with cutaneous involvement was that of a homogenous mass (Fig. 3). The mass exhibited iso-attenuation with the adjacent muscle and extended into the subcutaneous tissues, bones, muscles and mediastinum, with ill-defined margins. On MRI, KHE lesions typically appeared as heterogeneous hyperintense masses. Mild to moderate pericardial effusion was common. Other common imaging findings were emphysema, pneumonia and mild to severe pleural effusion. All but one patient presented with a solitary lesion. In patient #8, an enhanced CT scan revealed multiple mixed sclerotic lesions in the left root of the neck, left posterior chest wall and superior mediastinum, compressing the left primary bronchus. The lesions diffusely infiltrated the vertebral bodies of T1 and T6 and the parallel ribs (Fig. 4a).

### Pathological findings

All patients with KLA underwent incisional biopsy during the diagnostic workup (Fig. 1d). The histologic characteristics of KLA were dispersed and poorly marginated clusters or sheets of spindled lymphatic endothelial cells, which accompanied malformed lymphatic channels (Fig. 1e). The lymphatic origin of the lesions was confirmed by immunostaining of the lymphatic endothelial cells for CD31 (Fig. 1f) and D2–40 (Fig. 1g). The histologic findings were consistent with the diagnosis of KLA.

Specimens were taken during the active phase of KMP in five patients with KHE. The histologic hallmark of KHE was infiltrating, defined, rounded and confluent nodules, which were composed of spindle endothelial cells (Fig. 4b). These spindle endothelial cells aligned to form lymphatic channels and slit-like vascular lumina containing extravasation of erythrocyte with hemosiderin deposits. Immunohistochemical studies showed that the tumor cells were positive for CD31 (Fig. 4c), CD34 (Fig. 4d) and D2–40 (Fig. 4e).

### Management

Treatment was multimodal in patients with KLA; all patients received at least one therapeutic modality, including corticosteroids, vincristine, sildenafil, and sirolimus. No single or combination medical treatment produced consistently reproducible responses, defined as the improvement of the symptoms, shrinkage of the lesion size and/or restoration of the hematologic parameters. All patients received additional procedural therapies, including thoracotomy, thoracoscopy, chest tubes, pericardiocentesis and/or splenectomy. In three patients, there was no improvement in symptoms and/or resolution of the thrombocytopenia. Two patients required frequent platelet infusion and fresh frozen plasma to manage their thrombocytopenia and coagulopathy. Patient #3 has been treated with combination therapy of prednisolone (2 mg/kg/d) and sirolimus (0.8 mg/m^2^ administered twice daily). However, his response was suboptimal. He was advised to undergo splenectomy after a multidisciplinary discussion of his disease and treatment plan.

All but one patient with KHE required multimodal therapy; monotherapy was usually not sufficient to treat KMP. In 3 patients, multiple agents were given in sequence. Since 2011, the treatment most commonly administered was sirolimus plus prednisolone. This treatment regimen was effective in providing a rapid improvement of the hematopoietic parameters. After 3–4 weeks of combination treatment, the prednisolone was tapered and discontinued within the following 3–4 weeks, while sirolimus was continued.

### Follow-up

The mean length of follow-up for the entire cohort was 4.5 years (range, 1.2 to 9.0 years). Liver function, blood parameters, coagulation function and imaging examinations (CT and/or MRI) were the modalities used to monitor patients. Two patients with KLA (patients #4 and 5) suffered severe respiratory distress and repeated hemothorax. They died from acute heart failure and/or disseminated intravascular coagulopathy. Significant symptom relief was recorded in one patient with KLA who underwent splenectomy (patient #3). There was evidence of significant improvements in the pulmonary interstitial opacities, interlobular septal thickening and pleural effusion (Fig. 1h). In one patient (patient #1), although vincristine and sirolimus improved the associated respiratory symptoms, the patient experienced enlargement of his tumor mass and persistent mild thrombocytopenia despite prolonged sirolimus treatment (Fig. 2c-e). One patient had more additional small lesions in the liver and spleen, although he had been free of the recurrence of thrombocytopenia.

One patient with KHE died as a result of severe thrombocytopenia and coagulopathy despite aggressive treatments, including high doses of corticosteroids. The remaining six patients showed improvement. The KHE lesions continued to regress after the initial response, with an associated concurrent relief of symptoms (Fig. 4f-h). Five patients had been successfully tapered off treatment. Neither regrowth of the KHE nor hematologic abnormalities was observed at the last follow-up.

## Discussion

The natural histories of KLA and KHE and the treatment regimens of these lesions remain unclear. This is a large number of single institutional KLA and thoracic KHE review study. Our review demonstrated that KLA shares overlapping patterns of clinical symptoms, anatomical location, imaging features and complications with KHE. The accurate diagnosis of KLA and intrathoracic KHE can be hampered by this nonspecific presentation, including indolent respiratory symptoms, thrombocytopenia, and coagulopathy.

KHEs are found in varied locations, and approximately 88% of the lesions have cutaneous manifestations. The extremities are the most common anatomic locations. Intrathoracic KHEs and multifocal lesions are extremely unusual [11]. Intrathoracic lesions have been identified in 3 and 10% of patients with KHE by two of the largest retrospective studies, both of which collecting data from more than 100 patients with KHE [1, 4]. However, multifocal or diffuse KHE, which simultaneously involves the thorax and abdomen, has not been reported. In contrast, all patients with KLA had intrathoracic disease, and cutaneous involvement was rare. Microcystic lymphatic anomalies involving the lung parenchyma and spleen were noted in KLA [2]. Retroperitoneal lesions and noncontiguous bone destruction were also common in patients with KLA. In the present study, four patients with KLA had initially been diagnosed with pulmonary infections but failed to improve after treatment with antibiotics, prompting further diagnostic imaging.

Approximately 70% of patients with KHE develop KMP [1, 4]. Consistent with previous studies, we found that all intrathoracic lesions were associated with KMP. Clinically, intrathoracic lesions tended to be more expansive and infiltrative, and were more likely to develop KMP [5–7]. KMP in KHE is a profound thrombocytopenia with coagulopathy resulting from intralesional platelet trapping, followed by the activation and consumption of platelets. It is conceivable that the abnormal lymphatic endothelial cells in KHE lesions can directly trigger platelet activation via the CLEC2/podoplanin pathway [12]. In patients with KHE, the platelet counts are often less than 30× 10^9^/L. It is now clear that KMP occurs with KHE and tufted angioma and not with infantile hemangiomas and congenital hemangiomas. However, thrombocytopenia and coagulopathy in KLA may overlap with KMP in KHE. Interestingly, we found that the thrombocytopenia in some patients with KLA was extremely severe, similar to that observed in patients with KHE [13]. Nonetheless, our findings suggest that the presentation of diffuse, infiltrating, intrathoracic vascular lesions (with or without extrathoracic lesions) with refractory thrombocytopenia and coagulopathy can make the diagnosis of KLA relatively straightforward.

Pathologically, KHE lesions are clearly vascular tumors. In contrast, KLAs are complex vascular anomalies and are considered to share the characteristics of both tumors and malformations [14]. In patient #2, despite aggressive treatment, the splenic lesion progressed from a focal growth pattern to a multifocal growth pattern. This phenomenon further supports the concept that KLAs exhibit neoplasm features. As malformations, KLAs are presumed to have been present since birth [2]. A recent study revealed that KLA endothelial cells appeared inactive in supporting the formation of the vascular network, whereas KHE cells had this capacity [15]. Histologically, these lesions have similar histological characteristics. Both KLA and KHE are positive for the lymphatic marker D2–40, although the presence of nodules of spindle endothelial cells forming slit-like vascular channels is unique to KHE. In addition, the spindle endothelial cell component in KHE is primarily solid, with more defined, confluent vascularized nodules with microthrombi, glomeruloid foci and fibrin, whereas in KLA, the growth is usually sparse with poorly marginated clusters [16]. A somatic activating *NRAS* variant (c.182 A > G, p.Q61R) has been identified in lesional tissues from 10/11 patients with KLA. The study also indicated that the *NRAS* variant (c.182 A > G, p.Q61R) was absent from KHE samples, thus providing a molecular mean to further differentiate the two entities [17].

Although patients with KHE showed a wide variation in severity, recent studies have demonstrated that sirolimus monotherapy produced the best response in patients with KHE without KMP [18]. In addition, sirolimus in combination with short-term prednisolone has been proven to be effective in the treatment of refractory or severe KHE with KMP [10]. In the present report, the patients with KHE demonstrated dramatic improvement after treatment with a combination of sirolimus and prednisolone. Similar to KHEs, KLAs are uncommon, and therefore, there is a lack of controlled trials. Clinically, the majority of patients with KLA needed a combination of procedural and medical treatments. Medical therapies, including vincristine, corticosteroids, interferon and sirolimus, have been recommended for the management of KLA. However, the standard protocols remain unclear because the responses to therapy are variegated and unpredictable [2, 9, 13, 19, 20]. Compared with KHE, KLA appears to be more refractory to medical therapies. Despite aggressive multimodal treatment, the mortality rate of KLA is notably high [2].

Splenectomy was performed in one of our patients with KLA due to his poor response to a combination treatment of sirolimus and prednisolone. Interestingly, this patient experienced notable improvement in his symptoms and complications after splenectomy. Most importantly, the amelioration of the interlobular septal thickening was maintained. In the report by Croteau et al. [2], splenectomy was performed in 3 patients with KLA, with two responding with the normalization of platelet levels. These phenomena raise the question of whether splenic lesions could have a stronger preactivating effect on platelets and therefore on KLA aggravation. It is conceivable that rapid advances in medical science and any information or advice on diagnoses, treatment durations, or surveillance guidelines will facilitate the development of important new approaches for the management of patients with KLA and KHE.

## Conclusions

There are some clinical and histological similarities between KLA and KHE. However, KLA seems to be a more complex, multifocal lymphatic anomaly, which is different from KHE with respect to behavior, radiologic imaging, histopathologic features and response to therapy. Information is sparse regarding the pathogenesis of these aggressive vascular anomalies and the mechanism or mechanisms underlying the associated thrombocytopenia and coagulopathy. Our study highlights the importance of multidisciplinary efforts in diagnosing and managing KLA and KHE. Given the rarity of KLA and KHE, large collaborative studies are essential to further identify effective treatment regimens for these life-threatening vascular anomalies.

## Additional file

Additional file 1 **Table S1.** Clinical Characteristics of patients with KLA or KHE. (DOCX 25 kb)

## Data Availability

The datasets used and/or analyzed during the current study available from the corresponding author on reasonable request.

## Abbreviations

CT: Computed tomography
FIB: Fibrinogen
KHE: Kaposiform hemangioendothelioma
KLA: Kaposiform lymphangiomatosis
KMP: Kasabach-Merritt phenomenon
MRI: Magnetic resonance imaging
