# Supplementary material for: Kaposiform lymphangiomatosis and kaposiform hemangioendothelioma: similarities and differences
Source: Orphanet J Rare Dis. 2019 Jul 5;14:165. doi: 10.1186/s13023-019-1147-9 (PMC6612206; doi:10.1186/s13023-019-1147-9)
Supplement: Supplementary file 1 — Table S1. Clinical Characteristics of patients with KLA or KHE. (DOCX 25 kb) [file 13023_2019_1147_MOESM1_ESM.docx]

Additional file 1: **Table S1** Clinical Characteristics of patients with KLA or KHE

| Cases | Sex | Age at the time of the presentation of signs and/or symptoms | Age at diagnosis | Location | Types of lesion | Major signs, symptoms, and/or complications | Treatments | Outcomes |
| --- | --- | --- | --- | --- | --- | --- | --- | --- |
| ***KLA*** |  |  |  |  |  |  |  |  |
| 1 | Male | 20.0 m | 66.0 m | Lung, vertebrae, rib, mediastinum | Diffuse | Respiratory distress, pneumonia, pericardial effusion, chylous pleural effusion, hemothorax,  bone destruction, decreased ROM, chronic pain | Thoractomy, pericardiocentesis chest tube, pericardial drain, vincristine, sirolimus plus corticosteroid | Worse |
| 2 | Male | 8.0 m | 31.0 m | Lung, mediastinum, mesentery, pancreas, spleen, liver | Diffuse, | Abdominal distention, vomiting | Vincristine plus corticosteroid, sirolimus | Worse |
| 3 | Male | 9.0 m | 13.0 m | Lung, spleen | Diffuse | Cough, respiratory distress, pneumonia, pericardial effusion, pleural effusion | Pericardiocentesis, sirolimus plus corticosteroid, splenectomy | Improved |
| 4 | Female | 18.0 m | 37.0 m | lung, vertebrae, rib, mediastinum | Diffuse | Fever, dry cough, dyspnea, pneumonia, pericardial effusion, hemothorax, AHF, DIC | Thoractomy, chest tube, pericardiocentesis vincristine plus corticosteroid | Death |
| 5 | Male | 31.0 m | 62.0 m | Lung, mediastinum, vertebrae, pelvis | Diffuse | Dyspnea, pneumonia, pericardial effusion, hemothorax, DIC | Chest tube, sirolimus, sirolimus plus corticosteroid | Death |
| 6 | Female | 72.0 | 108.0 | Mediastinum, vertebrae, retroperitoneum，mesentery, ilium, ischium, femur, right tight | Diffuse | Pneumonia, pleural effusion, chronic pain | Thoractomy, chest tube, laparotomy,  sirolimus, sildenafil | Stable |
| ***KHE*** |  |  |  |  |  |  |  |  |
| 7 | Male | Birth | 1.0 w | Chest wall, sternum, mediastinum | Focal, 9.0 × 9.0 cm | Enlarging mass, Respiratory distress, pneumonia, pericardial effusion | Corticosteroid | Improved |
| 8 | Female | 7.0 m | 11.0 m | Neck, axilla, mediastinum, vertebrae | Multifocal, (Max 6.0 × 2.0 cm) | Fever, dyspnea, pneumonia, obstructive pulmonary emphysema, pericardial effusion, bone destruction | Sirolimus plus corticosteroid | Improved |
| 9 | Male | 10.0 m | 14.0 m | Mediastinum | Focal, 5.0 × 3.0 cm | Pericardial effusion,  pleural effusion | Thoractomy, propranolol, vincristine, sirolimus plus corticosteroid | Improved |
| 10 | Male | 1.0 m | 3.0 m | Mediastinum | Focal, 8.0 × 5.0 cm | Respiratory distress, pericardial effusion,  pleural effusion | Thoractomy, chest tube, corticosteroid, corticosteroid plus vincristine | Death |
| 11 | Male | 3.5 m | 4.0 m | Chest wall, mediastinum | Focal, 15.0 × 10.0cm | Enlarging mass,  pleural effusion, pericardial effusion | Sirolimus plus corticosteroid | Improved |
| 12 | Female | Birth | 3.0 m | Chest wall, sternum, mediastinum, left shoulder, abdominal wall | Focal, 13.0 × 7.0 cm | Enlarging mass, respiratory distress,  pleural effusion | Corticosteroid, vincristine plus corticosteroid | Improved |
| 13 | Male | 7.0 m | 10.0 m | Neck, chest wall, sternum, mediastinum, abdominal wall | Focal, 12.0 × 8.0 cm | Enlarging mass | Sirolimus plus  corticosteroid | Improved |

^*^KLA: kaposiform lymphangiomatosis; KHE: kaposiform hemangioendothelioma; ROM: range of motion; AHF: acute heart failure; DIC: disseminated intravascular coagulopathy

Patient # 8, 9, 11 and 12 have been included in previous studies.
